# Pulmonary surfactant augments cytotoxicity of silica nanoparticles: Studies on an in vitro air–blood barrier model

**DOI:** 10.3762/bjnano.6.54

**Published:** 2015-02-20

**Authors:** Jennifer Y Kasper, Lisa Feiden, Maria I Hermanns, Christoph Bantz, Michael Maskos, Ronald E Unger, C James Kirkpatrick

**Affiliations:** 1 Institute of Pathology, University Medical Center Mainz, Langenbeckstr. 1, 55101 Mainz, Germany; 2 Fraunhofer ICT-IMM, Carl-Zeiss-Str. 18–20, 55129 Mainz, Germany

**Keywords:** air–blood barrier, cytotoxicity, inflammatory response, pulmonary surfactant, silica nanoparticles

## Abstract

The air–blood barrier is a very thin membrane of about 2.2 µm thickness and therefore represents an ideal portal of entry for nanoparticles to be used therapeutically in a regenerative medicine strategy. Until now, numerous studies using cellular airway models have been conducted in vitro in order to investigate the potential hazard of NPs. However, in most in vitro studies a crucial alveolar component has been neglected. Before aspirated NPs encounter the cellular air–blood barrier, they impinge on the alveolar surfactant layer (10–20 nm in thickness) that lines the entire alveolar surface. Thus, a prior interaction of NPs with pulmonary surfactant components will occur. In the present study we explored the impact of pulmonary surfactant on the cytotoxic potential of amorphous silica nanoparticles (aSNPs) using in vitro mono- and complex coculture models of the air–blood barrier. Furthermore, different surface functionalisations (plain-unmodified, amino, carboxylate) of the aSNPs were compared in order to study the impact of chemical surface properties on aSNP cytotoxicity in combination with lung surfactant. The alveolar epithelial cell line A549 was used in mono- and in coculture with the microvascular cell line ISO-HAS-1 in the form of different cytotoxicity assays (viability, membrane integrity, inflammatory responses such as IL-8 release). At a distinct concentration (100 µg/mL) aSNP–plain displayed the highest cytotoxicity and IL-8 release in monocultures of A549. aSNP–NH_2_ caused a slight toxic effect, whereas aSNP–COOH did not exhibit any cytotoxicity. In combination with lung surfactant, aSNP–plain revealed an increased cytotoxicity in monocultures of A549, aSNP–NH_2_ caused a slightly augmented toxic effect, whereas aSNP–COOH did not show any toxic alterations. A549 in coculture did not show any decreased toxicity (membrane integrity) for aSNP–plain in combination with lung surfactant. However, a significant augmented IL-8 release was observed, but no alterations in combination with lung surfactant. The augmented aSNP toxicity with surfactant in monocultures appears to depend on the chemical surface properties of the aSNPs. Reactive silanol groups seem to play a crucial role for an augmented toxicity of aSNPs. The A549 cells in the coculture seem to be more robust towards aSNPs, which might be a result of a higher differentiation and polarization state due the longer culture period.

## Introduction

Biological barriers of the human body which directly interface the external environment have, besides their actual physiological function, the vital task of protecting the body from external hazards. Examples of these barriers are the skin, the intestine or the alveolar region of the lung. Comparing these protective barriers among each other the air–blood barrier displays (with a thickness of about 2.2 µm) the thinnest barrier. This makes it an ideal portal of entry for pathogens or aspirated nano-sized particles (NPs). It comprises an epithelial layer directed to the alveolar lumen and a microvascular endothelial layer, which is exposed towards the vessel lumen [1]. Before aspirated NPs encounter this cellular air–blood barrier, they impinge on the protective alveolar surfactant lining layer (10–20 nm in thickness), that covers the entire alveolar surface [2]. It has already been shown that regardless of the NP surface properties they will be submerged in the aqueous phase of the alveolar lining layer after crossing the pulmonary surfactant layer [3–4]. Thus, in vitro studies focusing on cytotoxicity of NPs at and transport of NPs across this cellular air–blood barrier, must take into account that a prior interaction of NPs with pulmonary surfactant components will occur. Pulmonary surfactant comprises up to 90% phospholipids (phosphatidylcholines, phosphatidylglycerols) and up to 10% fatty acids, cholesterol and the crucial surfactant proteins A, B, C and D [5]. Subsequently, the immersion of NPs in the surfactant lining leads to a coating with surfactant components such as lipids or proteins [6]. It has already been shown for amorphous silica nanoparticles (aSNPs) that they will be entirely coated with a phospholipid bilayer [7]. Consequently, an impaired cytotoxicity and transport/translocation to other organs may be perceived due to this surfactant coating*.* Several in vitro studies on aSNP toxicity have already been conducted using simple as well as complex multicellular in vitro systems of the air–blood barrier [8–11]. In order to approach more closely the in vivo situation, it is essential to incorporate pulmonary surfactant into the experimental design. Recent studies have already stressed the importance of lung surfactant by investigating the toxicity of lung surfactant-coated carbon nanotubes on a complex in vitro culture model of the airway barrier [12–13].

Therefore, we explored in this study the impact of the pulmonary surfactant formulation Alveofact^®^ on the cytotoxic effect of amorphous silica nanoparticles (aSNPs) using in vitro mono- and complex coculture models (MC and CC) of the air–blood barrier. As alveolar epithelial cells we used A549 and ISO-HAS-1 as microvascular endothelial cells in a coculture model. To evaluate in what way and to what extent different aSNP-surface functionalisations play a role in their cytotoxicity following interaction with lung surfactant, we investigated and compared aSNPs with three different surface modifications (aSNP–plain, –NH_2_, –COOH).

## Material and Methods

**Nanoparticles:** Sicastar Red, which were already described in Kasper et al. [10–11] are monodisperse amorphous silica nanoparticles (aSNP specification: spherical, unporous, ρ = 2 mg/cm^3^) in aqueous dispersion with a nominal diameter of 70 nm. They are commercially available from micromod Partikeltechnologie GmbH, Rostock, Germany (http://www.micromod.de). The particles are loaded with a fluorophore, namely Rhodamin B (Ex: 569 nm, Em: 585 nm), which is covalently attached to the SiO_2_ matrix. Sicastar Red is available with several different surface modifications. To study the influence of surface properties, particles with plain silica surface (Si–OH/Si–O^−^) were compared to carboxy (–COOH)- and amine (–NH_2_)-modified silica nanoparticles. Particle size was determined using the method of dynamic light scattering (DLS). Thus, the reported sizes are z-weighted mean values of the hydrodynamic diameter. Particle diameters were measured in cell culture medium (RPMI 1640) and, for reference, in water (containing 2 mmol/L sodium bromide to guarantee optimum colloidal stability). Two time points were chosen: 0 (at 25 °C) and after 4 h incubation at 37 °C, representing the starting and end point of particle incubation. DLS analyses were performed using a Microtrac NANO-flex (with a 180° backscattering setup). As it involves the least assumptions about sample properties (i.e., about size distribution), the data analysis method "Monodisperse" was used for the evaluation of the measurements. The results are summarized in Table 1.

**Table 1:** Hydrodynamic particle diameters (*D*_h_) were measured in cell culture medium (RPMI 1640) and, for reference, in water (containing 2 mmol/L sodium bromide to guarantee optimum colloidal stability). Two time points were chosen: 0 and 4 h, representing the starting and end point of particle incubation.

	aSNP–plain		aSNP–NH_2_		aSNP–COOH	
	D_h_ [nm]	±SD [%]	*D*_h_ [nm]	±SD [%]	*D*_h_ [nm]	±SD [%]

NaBr (0 h)	63.6	22	62.6	23	65.5	26
NaBr (4 h)	63.5	13	61.0	11	62.8	10
RPMI (0 h)	68.2	11	64.8	10	69.5	11
RPMI (4 h)	66.2	25	62.1	13	69.0	28

These results are comparable to previously reported data for particles of this manufacturer [10]: No significant change in particle size could be detected for any of the particle types in water as well as in RPMI 1640 cell culture medium. Furthermore, no significantly different particle sizes were measured for any of the different surface modifications. As the particles exhibit comparable sizes and an identical agglomeration behavior, their only differentiating property is their surface chemistry. This makes the selected samples appropriate candidates for a comparison of the influence of the surface properties on particle toxicity.

In presence of Alveofact^®^ (Lyomark Pharma), large agglomerates of a few hundred nanometers in diameter were found in cell culture medium (data not shown). However, the analysis of the pure Alveofact^®^ dispersion revealed that agglomerates of the same size were already present without nanoparticles. This leads to the conclusion that the standard procedure proposed by the manufacturer for dispersing the freeze-dried surfactant mixture is not suitable to achieve solvation of the lipoproteins on the molecular level; even colloidally stabilized lipid/protein agglomerates are not reached. Furthermore, the study of the agglomeration behavior of silica nanoparticles in the presence of proteins is highly complex and requires the use of multi-angle dynamic light scattering instrumentation and sophisticated data analysis methods [14]. Therefore, it will not be discussed within the scope of this publication.

**Alveolar surfactant:** Alveofact^®^ is a commercially available (Lyomark Pharma) neonatal surfactant substitution and originated from bovine alveolar lavage. It is composed of surfactant proteins SP-B and SP-C as well as phospholipids. It was suspended in PBS (phosphate-buffered saline) at a concentration of 40 mg/mL.

**Cell culture:** ISO-HAS-1 (human microvascular endothelial cell line, originated from [15–16] and A549 (human lung carcinoma cell line) purchased from ATCC (CCL-185, Promochem, Wesel, Germany) were grown in RPMI 1640 with GlutaMax^TM^ supplement (Gibco 61870-010), 10% FCS and Pen/Strep (100 U/100 µg/mL) and cultivated at 37 °C, 5% CO_2_. ISO-HAS-1 and A549 were passaged every third day at a dilution of 1:3 and 1:6 until passage 50 and 35, respectively.

**Monocultures (MC) in experimental procedures:** Prior to seeding cells, the 96-well plates (TPP, Switzerland) or 8 well µ-slides (ibidi) were coated with 50/300 µL fibronectin for 1 h at 37 °C (5 µg/mL, Roche Diagnostics, Mannheim). The A549 cells were seeded with a density of 3.2 × 10^4^ cells/well on 96-well plates and 7.7 × 10^4^ cells/well on ibidi µ-slides (ibiTreat, tissue culture treated, #80826) in RPMI 1640 medium (Gibco) with GlutaMax^TM^ supplemented with 10% FCS and Pen/Strep (100 U/100 µg/mL) and cultivated at 37 °C, 5% CO_2_ for 24 h prior to NP exposure to a confluent cell-layer.

**The coculture (CC) model of the air blood barrier:** The coculture technique was performed as described by Hermanns et al. [17] with some alterations. HTS 24-Transwell^®^ filters (polycarbonate, 0.4 µm pore size; Costar, Wiesbaden, Germany) were coated on both sides with rat tail collagen type-I (12.12 µg/cm^2^, BD Biosciences, Heidelberg, Germany). ISO-HAS-1 cells (2.1 × 10^4^/well 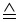 6.9 × 10^4^/cm^2^) were seeded on the lower surface of the inverted filter membrane. After 2 h of adhesion at 37 °C and 5% CO_2_, A549 (1.1 × 10^4^/well 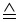 3.6 × 10^4^/cm^2^) were placed on the top side of the membrane. The cells were cultured for about 7 d in RPMI 1640 medium with LGlutaMax^TM^ supplemented with 5% FCS, Pen/Strep (100 U/100 µg/mL). From day 3 of cultivation the A549 were treated with dexamethasone (1 µM) until day 7. Due to the longer culture period of about 7 to 10 days Dex was necessary to form confluent and close epithelial and endothelial monolayers as it is seen for NCI H441/ISO-HAS-1 coculture in our previous studies [8–9,11]. Additionally, Dex directly suppresses effectively angiogenesis in endothelial cells [18]. Thus, these effects come in handy, since we focus on a confluent endothelial monolayer in the coculture. Dexamethasone definitely suppresses inflammatory responses of the cells. Therefore, to assure a putative inflammatory reaction of the coculture upon, i.e., nanoparticle exposure Dex was only administered during the culture period and was omitted during nanoparticle exposure.

**Nanoparticle and Alveofact^® ^application in cell culture:** The NP-application was conducted in the same manner as described in our previous studies [9–11]. NP-predilutions were prepared in pure water (Braun ad injectabilia, Braun Melsungen AG, Melsungen). All predilutions were applied 1:10 in serum-free medium to the cells (96er well and transwells: 10 µL NP-dispersion + 90 µL serumfree medium and ibidi wells: 30 µL NP-dispersion + 270 µL serum-free medium). Preliminary, cellular uptake of the NPs was examined for the monocultures of A549 on ibidi µ-slides. After NP-exposure for 4 h cells were washed with serum-free medium and cultured for further 20 h in fresh serum-containing cell culture medium. Subsequently, cells were fixed with methanol/ethanlol (2:1, rt, 20 min), washed 3 times with PBS and examined with a fluorescence microscope (Applied Precision, DeltaVision). To study cytotoxicity exposure times of 4 h were chosen and inflammatory responses were evaluated after 4 h/20 h (after 4 h incubation cells were washed twice with serum-free medium and further cultivated for 20 h period under normal cell culture conditions). To investigate the impact of Alveofact^®^ on aSNP toxicity, the surfactant has been applied and mixed thoroughly 1:10 in serum-free medium in the cell culture well prior to aSNP application (96er well and transwells: 80 µL serumfree medium + 10 µL surfactant suspension + 10 µL NP-dispersion). Endconcentrations of Alveofact^®^ in the well was 0.04 mg/mL.

**Cytotoxicity, determination of cell viability:** The viability of the cells was determined as described in our previous studies [9–11] using the CellTiter 96^®^ AQueous One Solution Cell Proliferation Assay (MTS, Promega, G3582). After nanoparticle incubation, medium was removed and cells were washed twice with PBS to remove nanoparticle remnants, which can interfere with the MTS-reagent. The MTS reagent (MTS stock solution mixed with medium in a ratio of 1:10) was applied to the cell layer for 45 min and transferred to a new plate to measure OD at 492 nm.

A quantification method for determination of the number of viable cells is cell staining with crystal violet (CV, purchased from Merck, 1407) [19]. Crystal violet (*N*-hexamethyl pararosaniline) is a monochromatic dye which stains cell nuclei. After fixation of NP-exposed cells they were incubated with 50 μL/96er well of a 0.1% crystal violet in aqua_dest_ solution for 20 min (rt, 70 rpm). Subsequently, the excessive dye was thoroughly washed away with tap water and dried over night at room temperature. Following this, cell-bound dye crystals were released with 100 μL 33% acetic acid for 10–15 min (rt, 70 rpm) and transferred to a new 96-well plate to measure the absorbance at 600 nm.

**Membrane integrity:** The membrane integrity was determined as described in our previous studies [9–11]. 25 µL of the supernatant, collected from nanoparticle-exposed A549 in mono- as well as coculture, were used in the LDH CytoTox 96^®^ non-radioactive cytotoxicity assay (Promega, G1780) to determine lactate dehydrogenase (LDH) release following membrane disruption after 4 h exposure. The NP-dispersions were checked for assay-interferences in regard to the absorbance readings with the NP-dispersion alone and in combination with the substrate reagent. No interferences occurred within the chosen NP-concentration range.

**Reactive oxygen species (ROS) production:** A549 cells were seeded in monoculture on 96-well plates as described in the section above (monocultures for experimental procedures). Prior to NP-exposure cells were incubated with the ROS detection reagent (10 µM in cell culture medium) 6-carboxy-2′,7′-dichlorodihydrofluorescein diacetate, di(acetoxymethyl ester) (Invitrogen, C2938) for 20 min at 37 °C and 5% CO_2_. Cells were washed twice with serum-free cell culture medium. As a positive control cells were incubated with 0.5 mM CoCl_2_ in parallel with the following NP-exposure in combination with Alfeofact^®^ as described above in section: Nanoparticle and Alveofact^®^ application in cell culture. Subsequent to the NP- and Alveofact-incubation period of 20 min, fluorescence was measured by means of a fluorescence spectrometer (Ex/Em wavelength 494/518 nm).

**Inflammatory responses:** As described in our previous studies [9–11] the supernatants were taken to determine IL-8 release via ELISA (DuoSet R&D, DY208) following the manufacturer’s recommendations.

**Immunofluorescence (IF):** Cells were fixed with paraformaldehyde (3.7%) in CS buffer (PIPES 0.1 M, EGTA 1 mM, 4% polyethylene glycol 800, NaOH 0.1 M) for 20 min at room temperature, and washed three times with PBS. Cell membranes were then permeabilized with 0.2% Triton X-100 in PBS for 10 min. After washing three times in PBS cells were stained with primary antibodies in PBS + 1% BSA for 1 h at room temperature (E-Cadherin, 1:100: Monosan, 7024; CD31; 1:40: Dako, M 0823). The secondary antibody (Alexa fluor 488-conjugated, 1:1000, anti-mouse, invitrogen) was added for 1 h after washing three times in PBS. Terminal cells were counterstained with 5 µg/mL Hoechst 33342 in PBS (Sigma, B 2261) for 5 min, washed 3 times with PBS and then mounted with Fluoromount-G™ (SouthernBiotech). Visual examination was conducted by means of a fluorescent microscope (personalDV, Applied Precision, Issaquah, USA).

## Results

**Figure 1:**
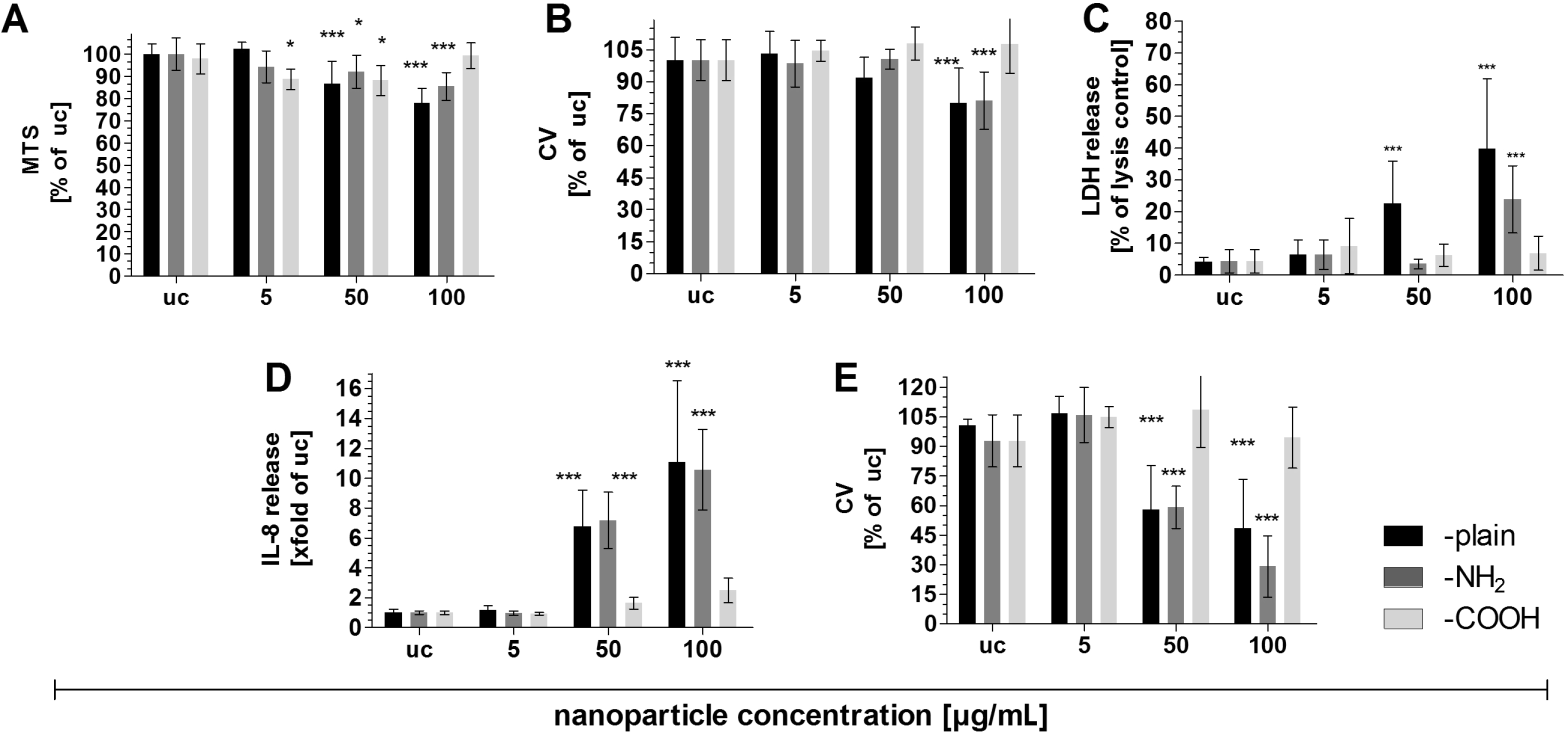
Comparison of cytotoxic effects on A549 after 4 h stimulation with aSNPs displaying different surfaces (–plain, –NH_2_, –COOH; 5–100 µg/mL, untreated control (uc)). A: Cell viability (mitochondrial activity, MTS, % of untreated control (uc)); B: cell viability (crystal violet staining of nuclei, cell loss measurement, % of uc); C: LDH release (membrane integrity, % of lysis control; D: IL-8 release after 4 h stimulation with 20 h further cultivation in fresh cell culture medium; E: cell viability (crystal violet, cell loss measurement) correlated to IL-8 (after 4 h stimulation with 20 h further cultivation in fresh cell culture medium). Data are depicted as means ± S.D. of 3 independent experiments with *n* = 3. For statistical analysis two-way ANOVA with Bonferroni’s post test was conducted. *P < 0.05, **P < 0.01 and ***P < 0.001 compared to the untreated control.

Figure 1A describes the results of the test for cell viability (mitochondrial enzyme activity, MTS) after 4 h aSNP exposure with different surface modifications (–plain, –NH_2_, –COOH). The aSNP–plain caused the highest toxic effect with increasing aSNP concentration (5 µg/mL: 102 ± 4.7% of untreated control (uc); 50 µg/mL: 86.5 ± 10% and 100 µg/mL: 77.9 ± 6.7%), with the first significant degree of toxicity being observed for a concentration of 50 µg/mL, increasing further with 100 µg/mL. Compared to the aSNP–plain, the surface-modified aSNP–NH_2_ (5 µg/mL: 94 ± 7% of uc; 50 µg/mL: 92 ± 7.4% and 100 µg/mL: 85 ± 6.3%) caused a slightly lower toxic effect. For the aSNP–COOH, however, a slight decrease of viability was observed for lower concentrations (5 µg/mL: 88.7 ± 4.6% of uc; 50 µg/mL: 88 ± 6.8%), but for 100 µg/mL (99 ± 5.7%) no significant decrease in viability was observed. In contrast to the MTS test, the viability assay using crystal violet (Figure 1B), did not detect a toxic effect for any of the concentrations of aSNP–COOH. Merely aSNP–plain and aSNP–NH_2_ showed a cell loss at a concentration of 100 µg/mL (80 ± 16% and 81 ± 13%). Figure 1C depicts the LDH (lactate dehydrogenase) release after aSNP treatment. The results obtained from this assay correlate with those of the MTS and crystal violet assays. The aSNP–plain caused the highest LDH release with increasing aSNP-concentrations (5 µg/mL: 6.5 ± 4.4% of lysis control; 50 µg/mL: 22.5 ± 13% and 100 µg/mL: 39 ± 22%). For the aSNP–NH_2_ only the highest concentration 100 µg/mL affected a LDH release comparable with a concentration of 50 µg/mL of aSNP–plain (23.8 ± 10%). For the aSNP–COOH no LDH release was observed for any aSNP-concentration. Figure 1D shows the IL-8 release after 4 h aSNP-incubation followed by 20 h further cultivation in fresh cell culture medium. A similar concentration-dependent inflammatory response is observed for aSNP–plain (5 µg/mL: (1.1 ± 0.2)-fold of uc; 50 µg/mL: (6.8 ± 2.4)-fold and 100 µg/mL: (11 ± 5.4)-fold) and aSNP–NH_2_ (5 µg/mL: (0.9 ± 0.1)-fold of uc; 50 µg/mL: (7.2 ± 1.9)-fold and 100 µg/mL: (10.6 ± 2.7)-fold). For aSNP–COOH a slight but non-significant (in comparison to aSNP–plain and –NH_2_) increase of IL-8 was detected for a concentration of 50 µg/mL ((1.6 ± 0.3)-fold of uc) and 100 µg/mL ((2.5 ± 0.8)-fold of uc). Figure 1E shows in correlation with the IL-8 release the cell loss after the 20 h further cultivation. A drastic cell loss was measured for aSNP–plain and –NH_2_ at a concentration of 50 µg/mL (58 ± 22% and 59 ± 10%) and 100 µg/mL (49 ± 25% and 29 ± 15%). No cell loss was observed after incubation with aSNP–COOH.

**Figure 2:**
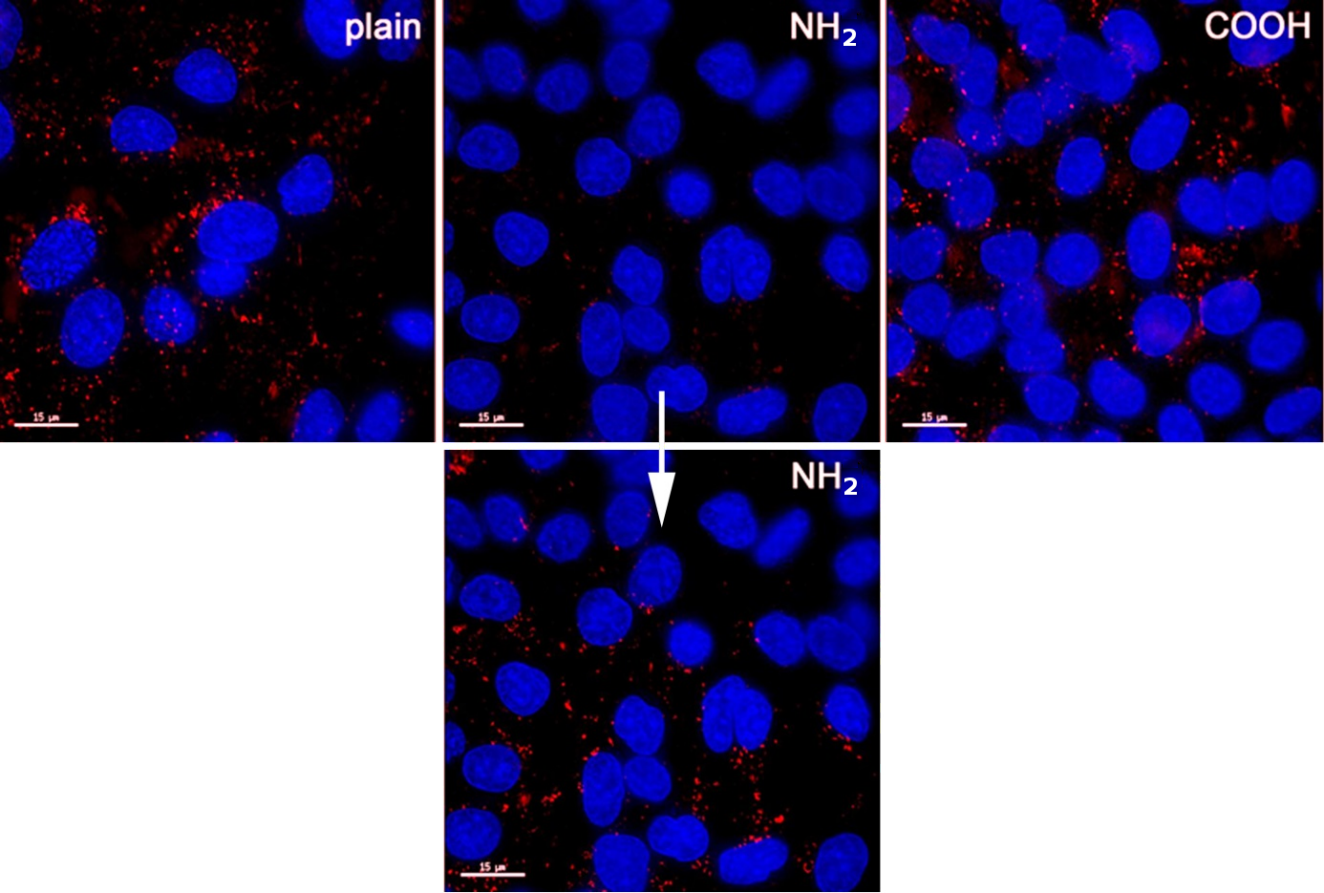
Cellular uptake of aSNPs with different surfaces in A549 (–plain, –NH_2_, –COOH; 50 µg/mL). The cells clearly internalized all three aSNPs after an incubation time of 4 h in serum-free medium. An approximate quantification of cellular uptake via fluorescence intensity measurement of the images could not be conducted due to the varying fluorescence intensities of the aSNP labeling itself. Comparing all three aSNPs using same intensity scale, a very low signal is observed for aSNP–NH_2_. However, after upscaling the intensity individually, a clear uptake is also observed for aSNP–NH_2_. Images were taken by a wide field fluorescence microscope (Applied Precision, DeltaVision), aSNP are displayed in red (496 nm), nuclei are stained with Hoechst 33433, scale bar 15 µm.

Figure 2 shows the cellular uptake of aSNPs with different surfaces in A549 (–plain, –NH_2_, –COOH; 50 µg/mL). The cells clearly internalized all three aSNPs after an incubation time of 4 h in serum-free medium. An approximate quantification of cellular uptake via fluorescence intensity measurement of the images could not be conducted due to the variable fluorescence intensity of the aSNP labeling itself. Comparing all three aSNPs using same intensity scale, a very low signal is observed for aSNP–NH_2_. However, after upscaling the intensity individually, a clear uptake is also observed for aSNP–NH_2_.

**Figure 3:**
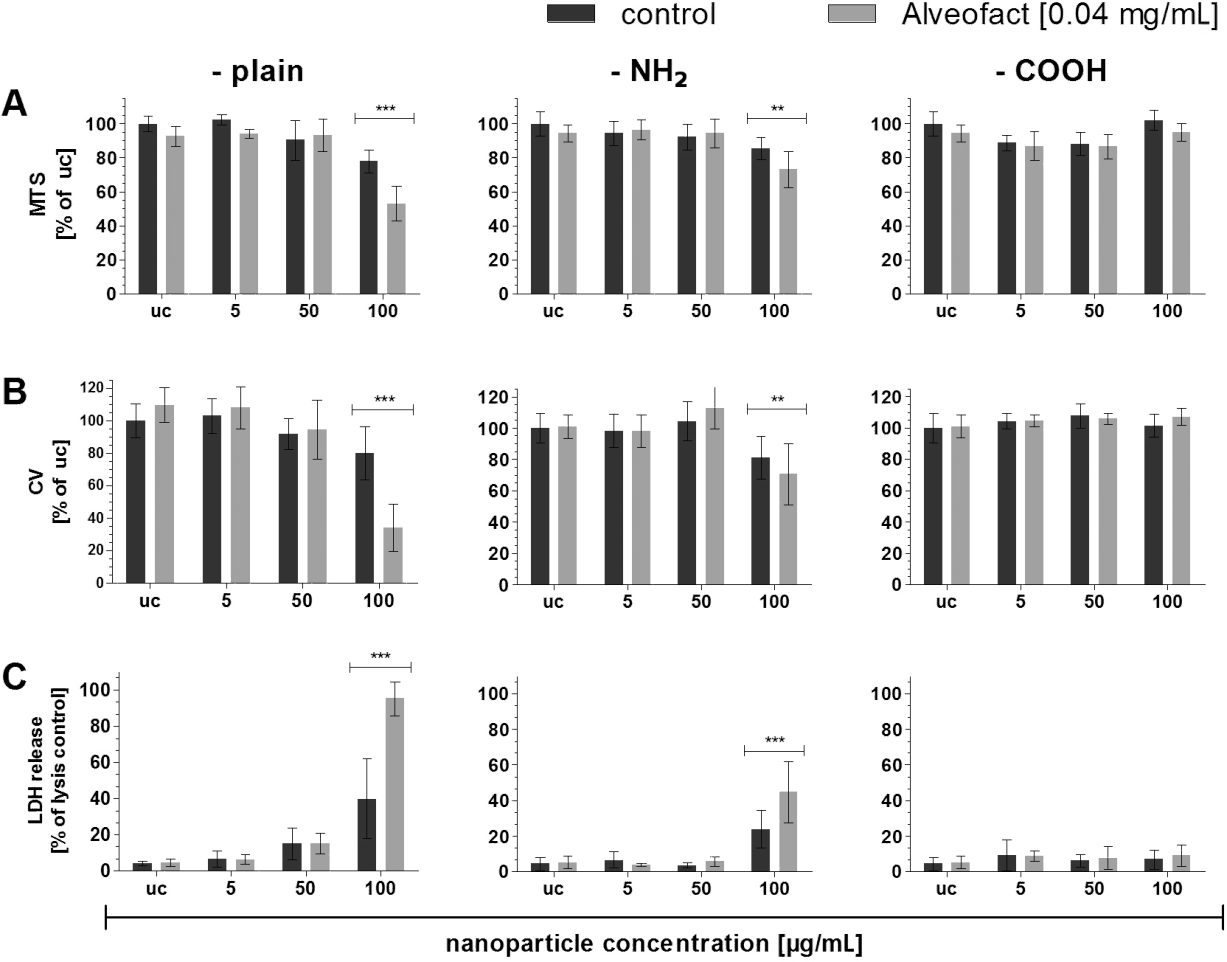
Comparison of cytotoxic effects on A549 after 4 h stimulation with different aSNPs (–plain, –NH_2_, –COOH; 5–100 µg/mL, uc: untreated control) in combination with (light grey columns) and without (dark grey columns) lung surfactant (Alveofact, 0.04 mg/mL). A: Cell viability (mitochondrial activity, MTS, % of uc); B: cell viability (crystal violet staining of nuclei, cell loss measurement, % of uc); C: LDH release (membrane integrity, % of lysis control). Data are depicted as means ± S.D. of 3 independent experiments with *n* = 3. For statistical analysis two-way ANOVA with Bonferroni’s post test was conducted. *P < 0.05, **P < 0.01 and ***P < 0.001 compared to the untreated control.

In Figure 3 the cytotoxicity of all three different aSNPs is compared in the presence or absence of lung surfactant (Alveofact^®^). The largest differences occurred for aSNP–plain at a concentration of 100 µg/mL. Cell viability decreased significantly after addition of 0.04 mg/mL Alveofact^®^ to the well at an aSNP–plain concentration of 100 µg/mL (without Alveofact^®^: 77.9 ± 6.7% of uc; with Alveofact^®^: 53 ± 10%). This result is further corroborated by the cell viability assay (crystal violet, without Alveofact^®^: 80 ± 16% of uc; with Alveofact^®^: 34 ± 14%)) and LDH assay (without Alveofact^®^: 39 ± 21% of lysis control; with Alveofact^®^: 95 ± 9%)). The combination of Alveofact^®^ with the aSNP–NH_2_ at a concentration of 100 µg/mL also caused a significantly higher toxicity, but the effect was lower compared to aSNP–plain. This was observed for all three toxicity assays: MTS (without Alveofact^®^: 85.4 ± 6.3% of us; with Alveofact^®^: 73 ± 10.5%), crystal violet (without Alveofact^®^: 81.4 ± 13% of us; with Alveofact^®^: 70.6 ± 19.4%) and LDH (without Alveofact^®^: 23.9 ± 10.5% of us; with Alveofact^®^: 45.6 ± 17%). However, there was no toxicity observed for the aSNP–COOH in combination with Alveofact^®^ in all three assays. Inflammatory responses (IL-8 release) were triggered in A549 after stimulation with aSNP–plain and –NH_2_ at a concentration of 50 µg/mL ((6.8 ± 2.4)-fold of uc; (7.2 ± 1.8)-fold) and 100 µg/mL ((11 ± 5.5)-fold; (10.5 ± 2.7)-fold).

**Figure 4:**
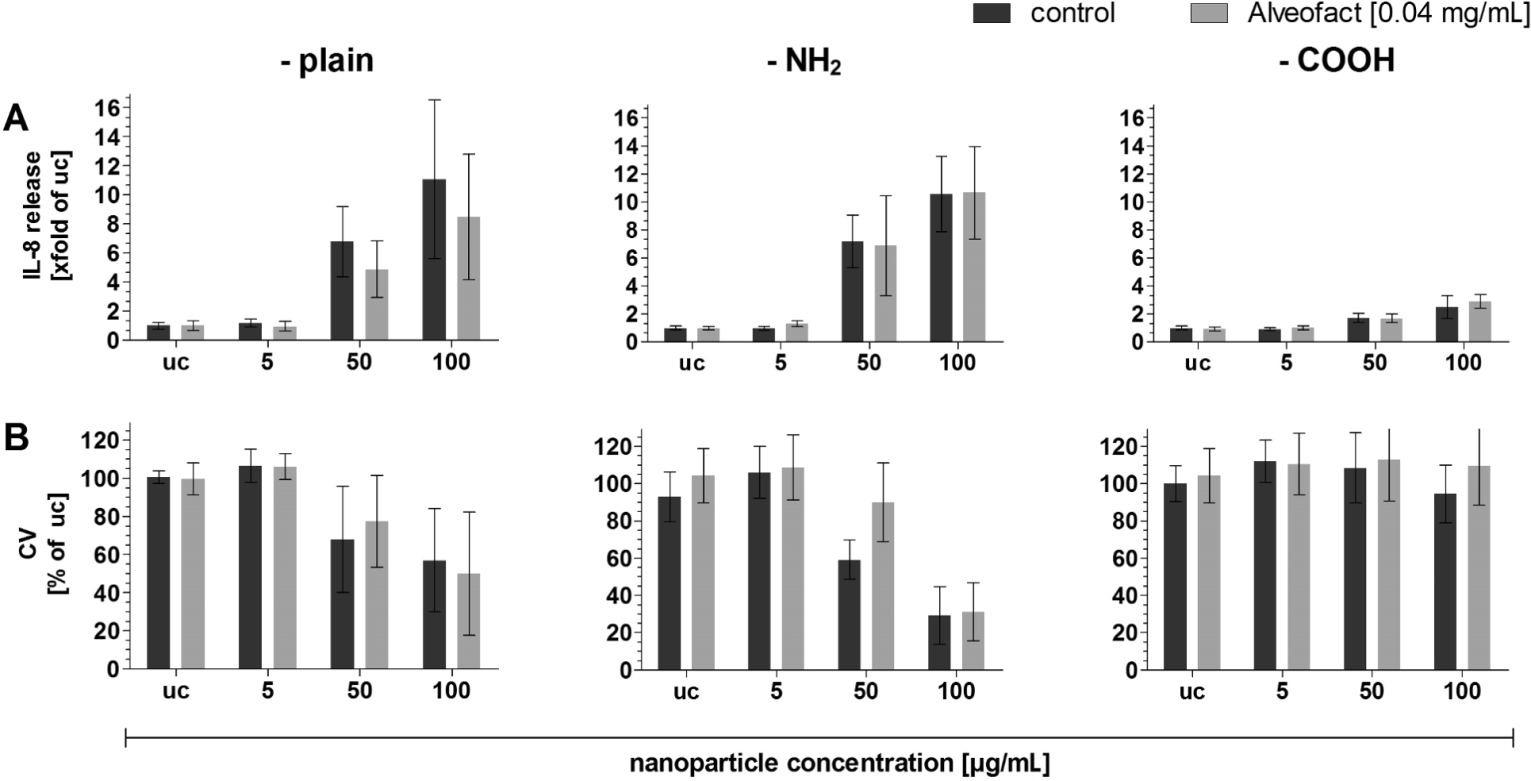
Comparison of inflammatory responses of A549 after stimulation with different aSNPs (–plain, –NH_2_, –COOH; 5–100 µg/mL, uc: untreated control) in combination with (light grey columns) and without (dark grey columns) lung surfactant (Alveofact, 0.04 mg/mL). Alveofact and aSNPs were simultaniously stimulated for 4 h and cells were then cultivated for a further 20 h period in fresh cell culture medium (4 h/20 h). A: IL-8 release after 4 h/20 h stimulation; B: cell viability (crystal violet, cell loss measurement) correlated to IL-8 (after 4 h/20 h). Data are depicted as means ± S.D. of 3 independent experiments with *n* = 3. For statistical analysis two-way ANOVA with Bonferroni’s post test was conducted. *P < 0.05, **P < 0.01 and ***P < 0.001 compared to the untreated control.

The combination of Alveofact^®^ with aSNP–plain and –NH_2_ stimulation did not result in a significant alteration of the aSNP-triggered IL-8 level (Figure 4A) regarding the toxic aSNP concentrations 50 µg/mL (–plain: (4.9 ± 2)-fold; –NH_2_: (6.8 ± 3.5)-fold) and 100 µg/mL (–plain: (8.5 ± 4.3)-fold; NH_2_: (10.7 ± 3.3)-fold). Figure 4B shows the viability assay crystal violet (cell number measurement) after the 20 h recovery period in fresh medium to correlate the IL-8 release to the actual cell number. The toxic concentrations for aSNP–plain and –NH_2_ 50 µg/mL (68 ± 28% and 59 ± 10.6% of uc) and 100 µg/mL (57 ± 27% and 29 ± 15% of uc) caused a significant cell loss, the addition of Alveofact^®^ to the aSNP stimulation did not cause an altered cell loss for both aSNP–plain and –NH_2_ (50 µg/mL: 77.5 ± 24% and 90 ± 21% of uc; 100 µg/mL: 50 ± 32% and 31 ± 15% of uc).

**Figure 5:**
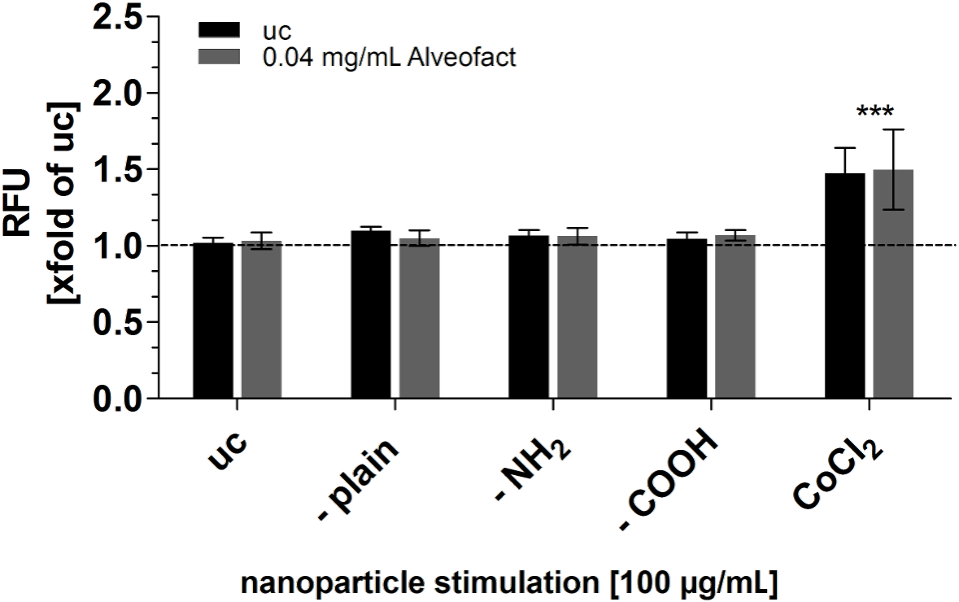
Reactive oxygen species (ROS) production in A549 after stimulation with different aSNPs (–plain, –NH_2_, –COOH; 5–100 µg/mL, untreated control (uc)) in combination with (grey columns) and without (black columns) lung surfactant (Alveofact: 0.04 mg/mL). Fluorescence intensity of the ROS detection reagent is depicted as relative fluorescence unit (RFU) related to uc. Positiv control: CoCl_2_ 0.5 mM. Incubation time of aSNPs and CoCl_2_ was 20 min, at which no cell loss occurred. Data are depicted as means ± S.D. of 3 independent experiments with *n* = 3. For statistical analysis two-way ANOVA with Bonferroni’s post test was conducted. *P < 0.05, **P < 0.01 and ***P < 0.001 compared to the untreated control.

Figure 5 illustrates the reactive oxygen species production after 20 min of aSNP stimulation (100 µg/mL) of A549 with and without Alveofact^®^. For all aSNPs in combination with or without Alveofact^®^ no cellular ROS cellular production could be detected. The positive control CoCl_2_ caused a fluorescence increase of the detection reagent C2938 (Invitrogen) of (1.47 ± 0.16)-fold of the untreated control (uc). An altered ROS production after CoCl_2_ stimulation in combination with Alveofact^®^ could not be observed ((1.49 ± 0.26)-fold of uc).

**Figure 6:**
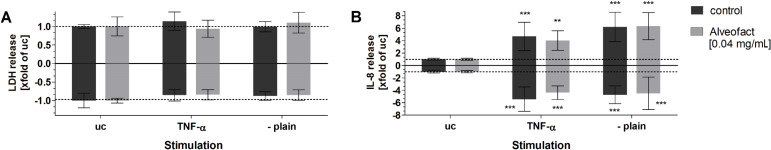
Cytotoxicity and inflammatory responses of aSNP–plain (100 µg/mL) in combination with alveolar surfactant (Alveofact (0.04 mg/mL), examined on the coculture model of the air-blood barrier A549/ISO-HAS-1. aSNPs were apically (A549) applied simultaneously with Alveofact^®^ in serum-free cell culture medium. A: LDH release was measured after 4 h aSNP incubation (membrane integrity, as xfold of untreated control with a previous normalization to the maximum release (lysis control = 100%)). Cells were then washed and cultured for further 20 h in fresh serum-containing cell culture media (4 h/20 h). B: IL-8 release was measured after 4 h/20 h and depicted as xfold of untreated control (uc). Upper columns: apical compartment, lower columns: basolateral compartment for both LDH and IL-8. Positive control: TNF-α 300 U/mL. Data are depicted as means ± S.D. of 3 independent experiments with *n* = 3. For statistical analysis two-way ANOVA with Bonferroni´s post test was conducted. *P < 0.05, **P < 0.01 and ***P < 0.001 compared to the untreated control.

In Figure 6 the LDH and IL-8 release of the coculture A549/ISO-HAS-1 is depicted after apical (A549) stimulation with aSNP–plain (100 µg/mL) in combination with and without Alveofact^®^. No LDH release could be measured either in the apical or in the basolateral compartment after aSNP–plain treatment for 4 h. Furthermore, no differences could be seen following the addition of Alveofact^®^. After 20 h recovery in fresh medium a significant production of IL-8 was observed for the aSNP–plain stimulated cocultures, although they demonstrated a similar behavior with (apical: (6.3 ± 2.2)-fold; basolateral: (4.5 ± 2.6)-fold of uc) and without Alveofact^®^ (apical: (6.2 ± 2.4)-fold; basolateral: (4.7 ± 1.4)-fold of uc).

**Figure 7:**
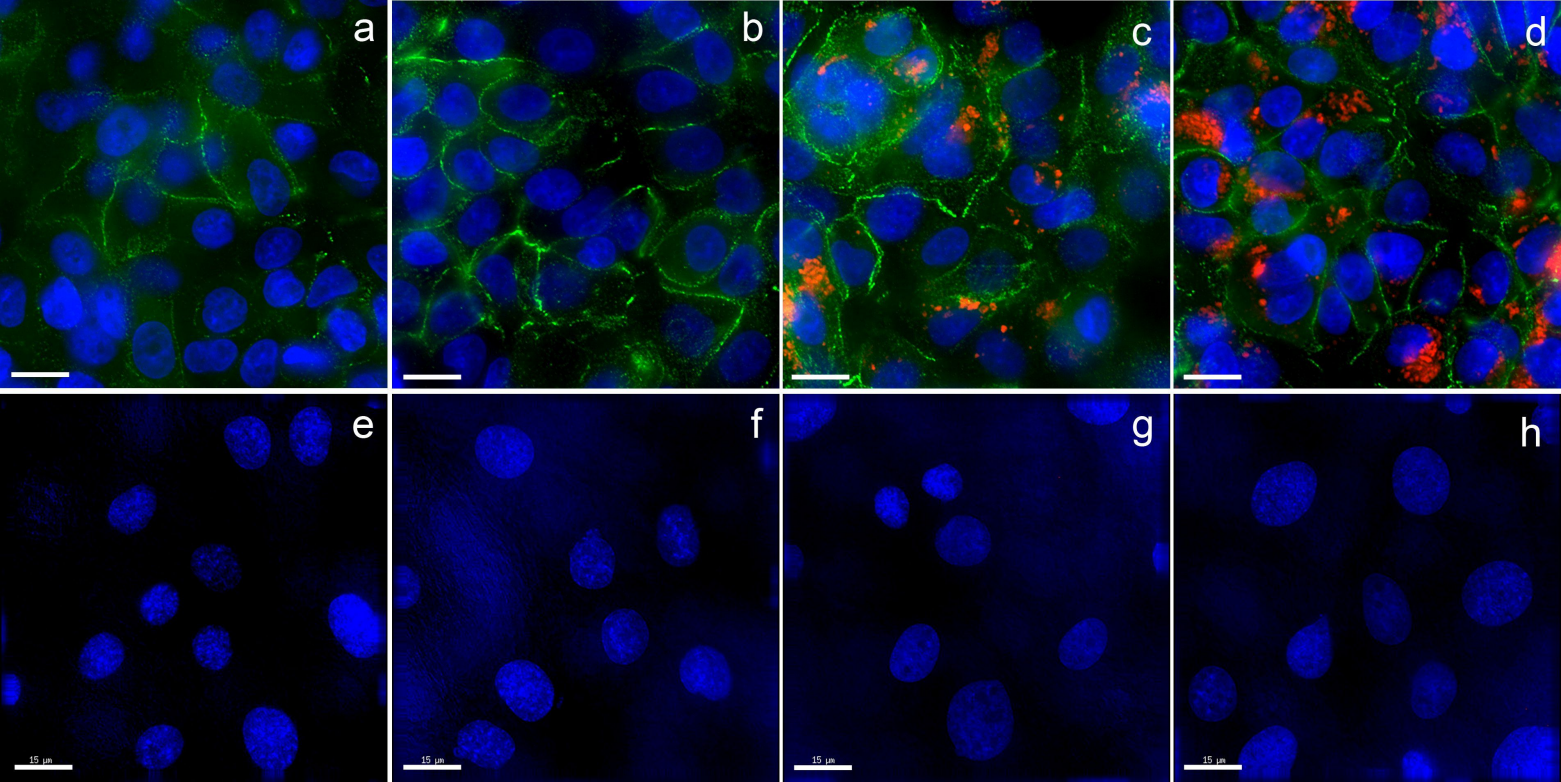
Cellular uptake of aSNP–plain (100 µg/mL, 4 h/20 h) in combination with Alveofact^®^ (0.04 mg/mL) was examined in the coculture of A549 (A–D) with ISO-HAS-1 (E–H). Green signal: E-cadherin counterstaining of A549 (a–d); red signal: uptake of aSNP–plain, which was apically applied to the A549. A/E: control; B/F: A549 treated with Alveofact^®^; C/G: A549 stimulated with aSNP–plain; D/H: A549 stimulated with aSNP–plain in combination with Alveofact^®^. E-cadherin staining of A549 shows an inconsistent pattern, although A549 as well as ISO-HAS-1 formed a confluent monolayer. No morphological differences could be observed for A549 and ISO-HAS-1 after stimulation with aSNP–plain, Alveofact^®^ or both. A clear uptake of aSNP–plain was observed with and without Alveofact^®^ treatment in A549, whereas no visual differences could be identified. No aSNP uptake could be detected in ISO-HAS-1, thus negating a transport of aSNPs through stimulated A549 according to this experimental setup. Pictures are taken by a wide field fluorescence microscope (Applied Precision, DeltaVision), nuclei are stained with Hoechst 33433, scale bar 15 µm.

Figure 7 shows the cellular uptake of aSNP–plain (100 µg/mL, 4 h/20 h) in combination with Alveofact^®^ (0.04 mg/mL) for the coculture A549/ISO-HAS-1. E-cadherin counterstaining (IF) of A549 is shown in Figure 7a–d. aSNP–plain, which was apically applied to the A549, is depicted in red. ISO-HAS-1 underneath aSNP–plain stimulated A549, (Figure 7e–f) were counterstained (IF) for CD31 (Figure 7f). E-cadherin staining of A549 shows an inconsistent pattern. However, A549 as well as ISO-HAS-1 formed a confluent monolayer. No morphological differences could be observed for A549 and ISO-HAS-1 after stimulation with aSNP–plain, Alveofact^®^ or both. A clear uptake of aSNP–plain can be observed with and without Alveofact^®^ treatment in A549, whereas no visual differences could be identified. According to this experimental setup no aSNP uptake could be detected in ISO-HAS-1, thus negating a transport of aSNPs through stimulated A549 and a subsequent uptake in ISO-HAS-1.

## Discussion

In this study, we investigated the influence of lung surfactant on possible cytotoxic effects of aSNPs using in vitro mono- and complex coculture models (MC and CC) of the air–blood barrier. aSNPs with three different surface modifications (aSNP–plain, –NH_2_, –COOH) were compared to investigate the influence of surface properties. In MC of A549 aSNP–plain displayed the highest cytotoxicity. aSNP–NH_2_ caused a lower toxic effect compared to aSNP–plain. Both showed a dose-dependent cytotoxicity. aSNP–plain was expected to cause the highest toxicity due to its reactive silanol groups as already reviewed by Napierska et al.*,* who stated that silanol groups directly cause cellular membrane hydrolysis [20]. According to the current literature, aSNPs with a positively charged amino-functionalisation regularly displayed higher cytotoxicity (compared to the carboxyl functionalization), while the negatively charged carboxyl-functionalisation proved to be mostly non-toxic [21–23].

NPs with a positively charged surface are usually applied in nonviral gene transfection and delivery studies using mainly cationic polymers or liposomes [8,24]. The positive NP surface charge enables better cellular contact and/or uptake than negatively charged or neutral molecules [25]. Nevertheless, the use of these positively charged drug and gene delivery carriers remains limited on account of their cytotoxicity. The cytotoxicity could be clearly related to the primary amine groups on the NP surface as reported previously by Agashe et al. [26]. A masking of the amine groups via further modifications caused a reduced toxicity of those NPs [27]. As discussed by Fröhlich et al., not only silica, but also ZnO, hollow silica-titania, and gold particles with a positive surface charge caused a higher cytotoxicity than the respective negative or uncharged NPs [24]. Likewise, carboxylic-modified silica nanoparticles, which have a negative surface charge, are of great interest as drug delivery vehicles for the controlled release of drugs [28–29]. However, others have shown that the toxicity of functionalized NPs is cell type-dependent, as they were able to demonstrate that carboxyl-functionalized NPs caused toxic effects in a macrophage cell line, RAW264.7, and a human embryonic kidney cell line, HEK293 [30]. By contrast, in the present study the aSNP–COOH showed a minimal decrease of mitochondrial activity in the alveolar epithelial cell line A549 at lower aSNP-concentrations (5–50 µg/mL), although no MTS-decrease was detected for the highest concentration (100 µg/mL). Nevertheless, no significant cell loss (CV), decrease of membrane integrity or increase of IL-8 release occurred for these concentrations of aSNP–COOH. As shown in Figure 2 the cellular uptake of all three aSNPs appears approximately equal as judged visually in individually optimized images. However, even an approximate uptake quantification via fluorescence intensity measurements was not suitable due to the different fluorescence-labeling intensities of the aSNPs themselves. The decrease of MTS conversion at lower concentrations of aSNP–COOH remains unexplained at the moment. The fact that in this study no cell loss or LDH release could be detected may indicate that metabolic function might be compromised by aSNP–COOH. In any case, it has already been shown by Harush-Frenkel et al., that differently charged NPs may have partially different endocytotic destinies. A small fraction of anionic NPs was retained within the endosomal system [31]. This may lead to altered metabolic cell functions, since endosomal sorting and trafficking is energy dependent. As discussed by Panariti et al.*,* NPs may cause either cell death or less serious side effects, both situations being normally taken together as “cytotoxicity” [32]. In our previous investigations regarding aSNP cytotoxicity the MTS assay correlated well with cell death, and was corroborated as cell loss in the crystal violet assay (CV), but not for lower concentrations of aSNP–COOH. However, no toxic effects were observed for a concentration of 100 µg/mL. Testing for any form of aSNP-assay interference did not reveal any false-positive results. A speculative explanation could be that the slightly but in this setup not significantly elevated IL-8 level as an accompaniment of augmented metabolic processes.

Subsequent to the examination of the cellular behavior of these three different functionalized aSNPs, lung surfactant was added to the NP-stimulation of A549. As described in Figure 3 the addition of lung surfactant components such as Alveofact^®^ (Lyomark Pharma) increased the cytotoxicity drastically after 4 h for aSNP–plain and slightly for aSNP–NH_2_ at a concentration of 100 µg/mL. No toxicity was observed for aSNP–COOH in combination with Alveofact^®^. These findings were corroborated by all three cytotoxicity assays (MTS, CV and LDH). However, an alteration of the IL-8 release as a result of the addition of lung surfactant could not be observed for aSNP–plain and –NH_2_. Interestingly, a similar toxicity pattern, which was detected for aSNP–COOH (decreased mitochondrial activity for lower aSNP–COOH concentrations) was also observed in combination with Alveofact^®^.

Already 24 years ago researchers considered a direct interaction of the reactive silanol groups with the cellular plasma membrane [33]. A bonding of these silanol groups to polar phospholipid headgroups of the plasma membrane, leads to membranolysis [34–35]. As formerly reported, bonding of aSNPs to dipalmitoylphoshatidylcholine (DPPC, phospholipid of the plasma membrane) disrupts its inter-head groups, which causes a higher mobility of the N(CH_3_)_3_^+^ group terminus of the phospholipids. Consequently, silica alters the membrane permeability and the fluidity of the bilayers is decreased, which finally leads to membrane perturbation and disruption [36]. The latter can be sensed by the Nalp3 inflammasome, thus initiating the release of pro-inflammatory cytokines [37]. Phospholipids such as DPPC are also a crucial component of lung surfactant. Other studies reported that the aSNP-silanol-phospholipid interaction or even aSNP in aqueous solutions have the potential to initiate directly the production of reactive oxygen species. This would explain the increased toxicity for aSNP–plain in combination with lung surfactant, but also for aSNP–NH_2_, taking into account the zeta potential of all three aSNPs (aSNP–plain: −23.4 mV; –NH_2_: −24.6 mV and –COOH: −29.3 mV, data kindly provided by the manufacturer micromod GmbH). aSNP–NH_2_ retained a negative “netto” surface charge according to their zeta potential, although it is supposed to display a positive charge on the basis of the amino-groups. This phenomenon was already described by Tenzer et al.*,* who concluded that according to the zeta potential the functionalization of similar aSNPs with amino groups was not saturated so as to mask negative charge of the surface silanols [38]. In terms of chemistry aSNP–NH_2_ and –COOH have remaining silanol groups to a similar extent. Since aSNP–COOH (with a similar amount of free silanol groups compared to aSNP–NH_2_) did not cause a toxic effect at 100 µg/mL this indicates that amino groups may also be able to interact with lung surfactant and hereby augment cytotoxicity.

With respect to possible mechanisms, the interaction of the silanol groups of aSNP–plain with lung surfactant might initiate extracellular ROS production or could trigger a cellular ROS production following internalization of the aSNPs. Extracellular ROS production likewise causes membranolysis [33,37,39–40]. To verify this hypothesis aSNP-stimulated A549 with and without Alveofact^®^ was checked for ROS over a period of 20 min to 24 h. However, no ROS could be detected after aSNP-stimulation regardless of the presence or absence of Alveofact^®^. The applied ROS detection assay (C2938, 6-carboxy-2’,7’-dichlorodihydrofluorescein diacetate, di(acetoxymethyl ester)) only detects intracellular ROS production, as occurs for the stimulation of A549 with CoCl_2_ (positive control). CoCl_2_ is often used as a hypoxia mimetic agent that triggers intracellular ROS generation [41]. Thus, an extracellular ROS production due to the silica particles interacting with the cell culture medium could not be directly verified by this assay. However, silica-produced extracellular ROS such as H_2_O_2_ itself triggers intracellular ROS production in the cells, since it is used as a positive control in many ROS detection assays. Thus, this indirectly negates extracellular ROS production due to aSNPs. Furthermore, aSNPs may internalize to a higher extent or use a different pathway, such as the lung surfactant recycling pathway, and therefore cause a higher toxicity. An internalized fraction of lung surfactant can follow a lysosomal degradation process in which potentially unmasked aSNP-silanol-groups could interact with phospholipids of the lysosomal membrane, which subsequently leads to lysosomal membranolysis, whereas proteolytic enzymes, which are released in the cytosol, could lead to cell lysis [37].

Surprisingly, the reduced membrane integrity (increased LDH-release), which was observed for A549 in MC after aSNP–plain (100 µg/mL) stimulation, could not be verified for the A549 in CC with or without Alveofact^®^. The culture conditions of MC and CC itself are different. The MC of A549 is seeded 24 h prior to the experiment, whereas the A549 in CC have a further 7 day period to develop a higher differentiation and polarization state, in which the plasma membrane composition reaches a higher protective function. This protective function may be exhibited by altered chemical interactions of the cellular membrane with the NP surface. These findings corroborate the hypothesis concerning a direct perturbation of the plasma membrane due to aSNPs. On the other hand endocytotic mechanisms may be different in the more differentiated CC compared to MC, thus cellular uptake behavior and the amount of internalized NPs in A549 may be different in both culture conditions. Nevertheless, a high IL-8 release is observed after aSNP–plain stimulation with and without Alveofact^®^ to a similar extent, which addresses the hypothesis of a minor membrane perturbation/ disruption, which is sensed by the Nalp3 inflammasome, initiating the release of pro-inflammatory cytokines such al IL-1β, followed by IL-8 [37]. Again, these observations suggest that inflammatory responses such as IL-8 release are a more sensitive indicator for sophisticated cell culture models, which mimic more closely the in vivo situation than do conventional cellcultures, as already discussed in former studies [9–11].

Cellular uptake of aSNP–plain by A549 in the coculture system with and without Alveofact^®^ did not reveal any differences as judged visually. Cellular aSNP uptake was observed for both experimental situations. After exposure of A549 to aSNP–plain (100 µg/mL) a transport of particles across the monolayer of stimulated A549 and subsequent uptake in ISO-HAS-1 could not be verified on the basis of the fluorescence microscopic images. It is well known that NPs are able to cross the air–blood barrier and reach various secondary organs [42]. In the present experimental setup, the transport of aSNPs might occur at such a low level that they could not be detected by a fluorescence microscope. A549 does not develop a measurable transepithelial resistance (TER), which is in marked contrast to the coculture of NCI H441 and ISO-HAS-1 [8–9,11].

## Conclusion

In this study we describe an enhanced cytotoxicity of silica nanoparticles following the addition of lung surfactant in an in vitro model of the air–blood barrier. The augmented toxicity in combination with surfactant appears to depend on the chemical surface properties of the aSNPs. Reactive silanol groups, and possibly amino-groups, seem to play a crucial role for this augmented toxicity of aSNPs in combination with lung surfactant. In addition, a higher differentiation and polarization state of A549 as it occurs in the coculture resembles more closely the in vivo situation, as they seem to be more robust towards aSNPs with respect to their membrane integrity, but still sensitive regarding their inflammatory responsiveness. In this cell culture setup, the lung surfactant was applied to the cultures simultaneously with the aSNPs, giving a preliminary insight into the rapid promoting effect of lung surfactant on aSNP toxicity.

In order to simulate in vivo conditions more closely the next step would be to study the cytotoxicity of aSNP-surfactant interaction on cell cultures kept on the air–liquid interface (ALI). On ALI the epithelial cells develop a physiological surfactant monolayer as it occurs in vivo*.* Prospectively, the results are relevant for the field of regenerative medicine, in which nanoparticles could be used for drug and gene delivery via the lung, as they demonstrate that model systems in vitro must take into account the complexity of the air–blood barrier, including the possible transport-modulating effects of surfactant components.
